# Recurrent Painful Ophthalmoplegic Neuropathy and Oculomotor Nerve Schwannoma: A Pediatric Case Report with Long-Term MRI Follow-Up and Literature Review

**DOI:** 10.1155/2019/5392945

**Published:** 2019-09-25

**Authors:** Maria Giuseppina Petruzzelli, Mariella Margari, Flora Furente, Maria Carmela Costanza, Anna Rosi Legrottaglie, Franca Dicuonzo, Lucia Margari

**Affiliations:** Department of Basic Medical Sciences, Neurosciences and Sense Organs Department, University of Bari “Aldo Moro”, Bari, Italy

## Abstract

**Background:**

Recurrent painful ophthalmoplegic neuropathy (RPON), previously known as ophthalmoplegic migraine (OM), is an uncommon disorder with repeated episodes of ocular cranial nerve neuropathy associated with ipsilateral headache. The age of presentation is most often during childhood or adolescence. MRI has a central role in the assessment of the RPON, especially to distinguish orbital, parasellar, or posterior fossa lesions that mimic symptoms of RPON. Actually, oculomotor nerve tumors may be masquerade as RPON so that MRI follow-ups are required to detect the possibility of tumor etiology.

**Case presentation:**

We report a 16-year-old boy with a 7-year follow-up and multiple brain MRI data, previously diagnosed as OM. The last brain MRI, performed during an acute phase of oculomotor paresis with ipsilateral headache, showed a nodular lesion described as schwannoma of III cranial nerve. Then, we reviewed the literature on OM and RPON in pediatric age with a focus on brain MRI findings.

**Conclusions:**

This review highlights the important role of serial brain MRIs in the long-term follow-up of RPON, especially in the cases with childhood onset, in order to not delay the diagnosis of a possible oculomotor nerve schwannoma.

## 1. Introduction

In the 3^rd^ edition of *International Classification of Headache Disorders* (ICHD) (2018) has been introduced a chapter named “painful lesions of the cranial nerves and other facial pains” after a consensus between the International Headache Society (IHS) and the International Association for the Study of Pain (IASP) [1]. The IASP defines neuropathic pain (NP) as “pain arising as a direct consequence of a lesion or disease affecting the somatosensory system” [2]. There is increasing awareness about NP in pediatric chronic pain because there are fewer information available regarding the prevalence of NP in the pediatric population than adults [3], and the affected children experience significant physical, psychological, and social sequelae that affect not only themselves but also family and friends [4]. The chapter “painful lesions of the cranial nerves and other facial pains” includes the recurrent painful ophthalmoplegic neuropathy (RPON), a condition characterized by repeated attacks of paresis of one or more ocular cranial nerves (commonly the III nerve), with ipsilateral headache [1]. This disorder is extremely rare and can occur at any age, but the highest prevalence is in children under the age of 12 years, with the median age at onset about 8 years [5]. The diagnostical definition of RPON changed throughout the history of headache classifications. The 1^st^ edition (1987) of the ICHD had considered this condition as a migraine variant [6], but in the ICHD-II (2004), the disorder, called “ophthalmoplegic migraine” (with the quotes around “migraine” included), was classified in the group of cranial neuralgias and central causes of facial pain [7]. However, the International Headache Society again revised the classification in 2013, renaming the disorder “RPON” [8]. Brain magnetic resonance imaging (MRI) is essential in the assessment of RPON to exclude orbital, parasellar, or posterior fossa lesions. Gadolinium enhancement of the affected nerve or nerve thickening can be evident using MRI during an acute episode of RPON, whereas negative findings are common during the period of full recovery [1, 9, 10]. Moreover, few MRI studies show the presence of a schwannoma in some patients with RPON disease. Isolated schwannomas are extremely rare. They are benign peripheral nerve sheath tumors, with slowlygrowing, accounting for 6% to 8% of all intracranial tumors [11, 12]. They usually arise from the Schwann cell layer of the vestibular branch of the VIII nerve; otherwise oculomotor nerve schwannomas without neurofibromatosis are very rare, and only few cases with pediatric onset have been described in literature [11–13]. In the contest of this ongoing debate [14] about the classification of RPON, we presented a pediatric case report with a 7-year follow-up and multiple brain MRI data, previously diagnosed as OM. The last brain MRI during an acute phase of oculomotor paresis with ipsilateral headache showed a nodular lesion described as schwannoma of III cranial nerve. Then, we reviewed the literature on OM and RPON with a focus on brain MRI findings.

## 2. Methods of Review

An electronic literature search was conducted for all articles published up to December 2018, with a range of databases searched including PubMed, Scopus, and Web of Science. The following combinations of keywords were searched: “opthalmoplegic migraine AND magnetic resonance imaging” and “recurrent painful ophthalmoplegic neuropathy AND magnetic resonance imaging.” The initial search returned 227 records; we considered 117 publications after screening to remove duplication of papers from different sources of research. Research articles, case reports, and reviews describing patients under the age of 18 years have been included. As inclusion criteria papers needed to provide enough information to extract clinical features of headache and oculomotor involvement and a description of MRI findings, 28 publications for a total of 43 clinical cases were selected for inclusion in this review.

## 3. Case Report

**Figure 1:**
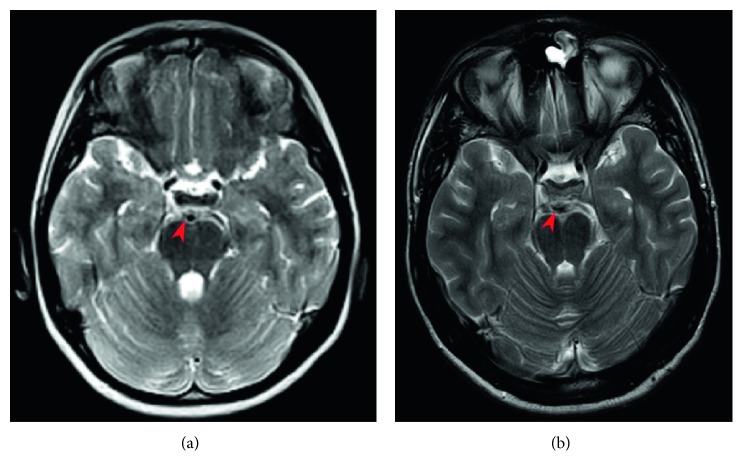
Axial T2-weighted MRI images comparison: (a) no focal or wide thickening of the oculomotor nerve during an acute attack of OM (Jan 2011); (b) 5-6 mm nodular mass suggesting an oculomotor nerve schwannoma (Oct 2018).

**Figure 2:**
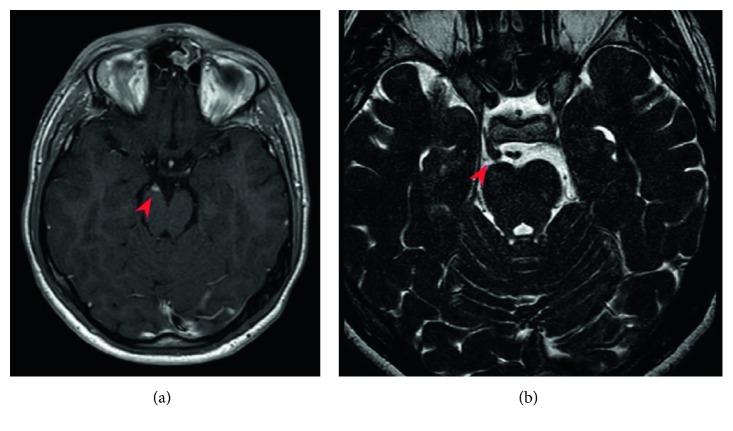
Axial T1-weighted (a) and axial T2-weighted (b) MRI images showing 5-6 mm nodular mass located within the fork of the right basilar artery near to perimesencephalic and interpeduncular cisterns (Oct 2018).

We reported the case of a male patient, 16 years old, with a history of frontal headaches accompanied by photophobia, nausea, and vomiting since the age of 4. He had his first episode of migraine associated with ocular pain and ptosis in the right eyelid, lasting 24 hours, in Jun 2010 at the age of 6. Six months later (Jan 2011), he had a heavier and persistent episode of migraine associated with right ocular pain (partial responsiveness to administration of paracetamol), right ptosis, and diplopia, so he was admitted to the Child Neuropsychiatry Unit, Department of Basic Medical Sciences, Neuroscience and Sense Organs, University of Bari “Aldo Moro,” Italy. Family history of migraine in both parents, multiple sclerosis in the father's family, and gastric neoplastic disease in the mother's family was referred. General physical examination was normal; neurologic examination showed paresis of the third and sixth cranial nerves. Laboratory investigation, including virological and organ-specific serum antibodies, and instrumental examination including awake/sleep electroencephalograph produced normal results. Brain and cervical-spine magnetic resonance imaging (MRI) enhanced after contrast administration and magnetic resonance angiography (MRA) produced normal findings. Diagnosis of “ophthalmoplegic migraine” was made according to ICHD 2, as discussed in a previous publication [10]. During the following years, about one time a month, the subject suffered from a moderate to severe intensity headache in the frontal and supraorbital region on the right side with a transient remission with paracetamol. Since his first hospitalization, he had 2 more episodes, in Jul 2013 and Jun 2016, of OM, with ocular pain ptosis in the right eyelid and diplopia. In both episodes brain MRI confirmed normal images. The diagnosis was revised as RPON according to the new diagnostic criteria of ICHD-3 beta (2013) [15]. At the age of 15 years (Sep 2018), the patient was again admitted to our Child Neuropsychiatry Unit manifesting moderate and persistent migraine, nausea, vomiting, phonophobia, and photophobia associated with right ocular pain, diplopia, and ptosis. Neurologic examination showed right eyelid ptosis, and ophthalmologic evaluation showed paresis of the III cranial nerve. Brain MRI with MRA revealed a 5-6 mm nodular enhancement mass, located within the fork of the right basilar artery near to perimesencephalic and interpeduncular cisterns, suggesting an oculomotor nerve schwannoma without any vascular malformation (Figures 1 and 2). The subject was treated with corticosteroid therapy because of the partial and transient responsiveness with paracetamol during acute episodes of right eye pain. He showed gradual and progressive improvement of headache symptoms in about one week.

## 4. Discussion

To our knowledge, this is the first case reported in the literature of an adolescent suffering from headache and ophthalmoplegia with a 7-year brain MRI follow-up, raising the question on the relationship between RPON and schwannoma. The evolutive changes in the presentation of symptoms and the different findings over time in brain MRI images observed in this patient repurpose some open issues on the classification and pathophysiological mechanisms of RPON. The patient had his first clinical manifestations of recurrent headaches at the age of 4 years agreeing to diagnostic criteria of migraine without aura. Since he was 7 years old, the patient started to have migraine episodes with the same clinical features but lasting more time and with partial responsiveness to administration of paracetamol with association of right ocular pain, ipsilateral ptosis, and diplopia. After two episodes with these features and with MRI that showed normal findings in the image scans without any appreciable enhancement or thickening of the nerve, he was diagnosed with “ophthalmoplegic migraine” according to ICHD-II [7]. Clinical and instrumental data at next follow-ups were in agreement with the reclassification of RPON proposed in the ICHD-3 [1]. Brain MRI follow-ups of 2013 and 2016 both during an acute episode of headache with ophthalmoplegia confirmed normal findings in the image scans in absence of any brain MRI data supporting a secondary cause. At this regard, according to our review, patients diagnosed with OM/RPON have MRI exams that show thickening and enhancing of the oculomotor nerve [9, 16–24] or normal findings in image scans [18, 20, 22, 23, 25–30]. Nevertheless, the last brain MRI examination of our patient showed the presence of 5-6 mm nodular enhanced mass located within the fork of the right basilar artery near to perimesencephalic and interpeduncular cisterns, enhanced after contrast, which could be described as III cranial nerve schwannoma (Figures 1 and 2). This case highlights the important role of longitudinal follow-up of brain MRI in RPON and raises the question on the relationship between RPON and schwannoma. The exact pathophysiology of RPON is unknown, but the most accepted postulated mechanisms include microvascular ischemia, demyelization, and inflammation involving the oculomotor nerve [31]. Despite the reclassification as a neuropathy, a balanced debate on arguments pro and contra a migrainous background of RPON have been published. Friedman DI proposed to consider OM as a syndrome that may be “primary” as a variant of migraine with aura for cases with normal imaging and spontaneous resolution and “secondary” as a cranial neuropathy with focal nerve enhancement on neuroimaging [31]. The case we described is far from this classification hypothesis, considering that three different brain MRIs during an acute attack of OM showed normal findings inducing to consider the clinical manifestation of the patient as a “primary” migraine, while the last brain MRI showed a focal enhancement of contrast, described as a schwannoma. The review of literature on MRI findings in child and adolescent patients affected by OM/RPON (according to ICHD classifications over time) showed only three cases with isolated schwannoma of the oculomotor nerve associated with migraine and oculomotor nerve palsy after a long time history of III nerve paresis and/or migraine (Table 1). In 2005, Murakami et al. published the first histologically proved case of schwannoma in an 11-year-old girl with a previous history of OM. The initial diagnosis of OM was corroborated by brain MRI. Five years later, the OM attacks became more frequent and not controlled by prophylaxis or acute episodes' drugs, and brain MRI with three-dimensional reconstruction shows a 5 mm nodular lesion highly suggestive of a neoplastic process involving the oculomotor nerve in its course from the brainstem through the prepontine cistern continuously. Histopathological finding after surgical treatment confirmed the diagnosis of schwannoma [35]. Riahi et al. in 2014 published the case of a 12-year-old girl who presented three episodes of left eye painful ophthalmoplegia during a period of 3 years. Two previous brain MRIs produced normal image scans, while the brain MRI made after the third episode showed a mass of tissue in the cavernous sinus suggesting a third nerve schwannoma [42]. Jibia et al. in 2015 published the case of a 13-year-old girl with a previous medical history of migraine since she was 6 years old. In coincidence of hospitalization because of an attack of disabling ophthalmic migraine, the brain MRI revealed a right nodular schwannoma located within the cisternal segment of the oculomotor nerve (patient was treated with analgesic and corticosteroid therapy with complete regression of symptoms three weeks later and a normal MRI follow-up) [43]. The most part of other cases included in our review, with OM/RPON diagnosis have short-term MRI follow-ups performed less than two years after the first evaluation that can show persistent thickening and enhancement [16, 17, 20–24] or their negativization [9, 18, 23]. Therefore, it is important to consider the long-term evolution of RPON and its relationship with oculomotor nerve schwannoma. Two different speculative explanations of the eventual pathophysiological relationship between RPON and schwannoma have been proposed. The first one is related to the intermittent release of a chemical substance from the tumor, which results in the stimulation of the trigeminal nerve receptors leading to a migrainous headache. According to this hypothesis, the oculomotor nerve schwannoma mimics RPON, so the painful ophthalmoplegia may be considered as the initial manifestation of a tumor and may be too small to be appreciated in MRI scans [35, 44]. A review of pediatric isolated oculomotor nerve schwannoma showed OM as one among the clinical signs of presentation of this tumor [11]. The alternative hypothesis is based on the idea that the underlying pathophysiology of RPON is an inflammatory cranial neuralgia and not migraine, so that repeated episodes of inflammation leading to demyelination and remyelination may be associated with a potential transformation into schwannoma from Schwann cells proliferation. According to this interpretation, isolated oculomotor schwannoma may be considered as a long-term complication of RPON.

## 5. Conclusions

In conclusion, both case reports we presented and the literature review of other pediatric cases of OM/RPON, suggest an existing relationship between RPON and schwannoma. Further longitudinal studies on a larger number of cases are needed to clarify whether in some patients an ocular motor nerve schwannoma may closely mimic RPON or, alternatively, if the RPON may be considered a risk factor to develop over time a schwannoma. Anyway, in both these situations, negative findings at MRI during an acute attack of RPON (without any thickening or gadolinium enhancement) do not rule out the possibility of a future evidence of the tumor, considering that schwannomas are generally slowly progressive. Serial brain MRIs during acute exacerbations and even between attacks are essential in the long-term follow-up of RPON, especially in the cases with childhood onset, in order to not delay the diagnosis of a possible oculomotor nerve schwannoma.

## Figures and Tables

**Table 1:** A literature review of symptoms and magnetic resonance imaging findings in 43 patients under the age of 18 years with ophthalmoplegic migraine or recurrent painful ophthalmoplegic neuropathy.

Author	Reference	Case	M/F	Age of onset	Age of observation	Headache	Oculomotor involvement	MRI	MRI follow-up	Diagnosis
Aers et al.	[16]	1	F	8	12	Severe right unilateral headache with photophobia and vomiting	Diplopia and ptosis of the right upper eyelid	MRI: marked thickening and gadolinium enhancement of the right oculomotor nerve along its subarachnoid course in the interpeduncular fossa	Six months later: slightly thickened but no longer enhancing cisternal portion of the right oculomotor nerve	OM
Wong and Wong	[32]	2	M	6	6	Acute onset of headache with photophobia and vomiting	Diplopia, acute periorbital pain with droopy eyelids	No contrast MRI scan: slight asymmetry in size of the oculomotor nerve	NA	OM
Mark et al.	[17]	3	F	Not reported	8	Headache	Oculomotor nerve palsy	Acute MRI: focal thickening and enhancement	7 to 9 weeks later: resolution of the enhancement	OM
Mark et al.	[17]	4	M	Not reported	12	Headache	Oculomotor nerve palsy	Acute MRI: focal thickening	7 to 9 weeks later: resolution of the enhancement	OM
Mark et al.	[17]	5	F	Not reported	5	Headache	Oculomotor nerve palsy	Acute MRI: focal thickening and enhancement	7 to 9 weeks later: resolution of the enhancement	OM
Mark et al.	[17]	6	M	Not reported	3	Headache	Oculomotor nerve palsy	Acute MRI: whole thickening and enhancement	7 to 9 weeks later: resolution of the enhancement	OM
Prats et al.	[18]	7	F	11	12	Throbbing headache	Defective elevation and adduction of the left eye and mild dilation of the left pupil and diplopia	MRI: enlargement of the cisternal portion of the left III cranial nerve along the first 3 mm of its course, unilateral enhancement after intravenous gadolinium administration	A second MRI with contrast administration: normal	OM
Prats et al.	[18]	8	M	3.5	5	Throbbing headache	Complete III cranial nerve palsy of the left side	MRI: enlargement and enhancement of the cisternal portion of the left III cranial nerve	40 days later: similar findings; 4.5 years later: normal	OM
Prats et al.	[18]	9	F	6	6	Headache in the left orbital region	Moderate left ptosis of the eyelid, mild mydriasis, and displacement of the eye upward and outward	MRI: normal	5 years later: normal	OM
Del Toro et al.	[33]	10	M	10	10	Ocular pain	Complete right third nerve palsy	MRI: right cavernous sinus enlargement, more obvious after intravenous gadolinium administration	1 month later: the normal caliber of the right carotid artery similar to the left with normal appearance of the ipsilateral cavernous sinus	Tolosa–Hunt syndrome
O'hara et al.	[21]	11	F	4	7	Acute onset of severe headache, nausea, and vomiting	Complete right III nerve palsy	Right parasagittal T1-weighted MRI scan postcontrast: enhancing cisternal portion of the right CN III with nodular mass-like thickening of the proximal portion adjacent to the brainstem	6 weeks later, follow-up postcontrast: marked improvement in enhancement and thickening of the right CN III	OM
O'hara et al.	[21]	12	M	2	3	Headache	Complete left CN III palsy	Left parasagittal T1-weighted MRI scan postcontrast: thickened enhancing cisternal portion of CN III	8 months later: coronal T1-weighted scan postcontrast again: mass-like thickening and enhancement of the proximal cisternal portion of the left CN III	OM
Lance and Zagami	[22]	13	F	3	16	Sharp pain behind the left eye, followed by drooping of left eyelid; nausea, vomiting, and sensitivity to light, sound, and smell	Left ptosis with paresis of upward deviation in the left eye which turned outwards as she attempted to look up	Acute MRI: enhancement of the intracisternal portion of the oculomotor nerve with gadolinium	Repeat MRI: enhancement of the oculomotor nerve present but less intense	OM
Lance and Zagami	[22]	14	M	5	5	Right-sided headache accompanied by vomiting	Drooping of right eyelid and difficulty in looking upwards and inwards with his right eye	Unenhanced MRI normal	NA	OM
Lance and Zagami	[22]	15	M	3	13	Left frontal headache, vomiting, and photophobia	Ptosis	Unenhanced MRI normal	NA	OM
Shin et al.	[25]	16	M	7	8	Severe, throbbing, left periorbital headache with associated nausea, vomiting, and photophobia	Diplopia and drooping of the upper left eyelid	Contrasted MRI: no abnormal findings	NA	OM
Shin et al.	[25]	17	F	6	11	Right periorbital headache	Drooping of the right eyelid and diplopia	Contrasted MRI: no abnormal findings	NA	OM
Weiss and Phillips	[26]	18	M	2	7	Left supraorbital pain, nausea, and vomiting	Left ptosis, exodeviation of the left eye with horizontal and vertical diplopia	Brain MRI scan: normal, no contrast enhancement of the oculomotor nerve at its exit from the midbrain	NA	OM
Farage et al.	[34]	19	M	16	16	Pulsatile migraine associated with nausea	Ptosis, mydriasis, and divergent strabismus	MRI weighted in T1 with contrast: enhanced signal at left oculomotor nerve in cisternal portion	18 months later: no remarkable lesion	OM
Huang et al.	[19]	20	F	9	9	Acute-onset right frontal and periorbital eye pain with migrainous characteristics	Ptosis of right eyelid, a dilated pupil with slow pupillary response to light and a extraocular motor weakness compatible with partial oculomotor palsy	MRI: thickening of the oculomotor nerve with abnormal enhancement at the exit of brainstem on the right side	NA	OM
Murakami et al.	[35]	21	F	4	6	Severe throbbing headache	Diplopia, left blepharoptosis, and nonreactive left midriasis	MRI after contrast: a thickened oculomotor nerve in its course from the brainstem through the prepontine cistern continuously	NA	Schwannoma
Choi et al.	[36]	22	F	13	13	Blurred vision, photophobia, and pulsating headache	Complete right side internal ophthalmoplegia	MRI: gadolinium enhancement on the cisternal portion of right oculomotor cranial nerve	8 months later: gadolinium enhancement at the proximal part of the left oculomotor nerve	OM
Bharucha et al.	[9]	23	F	1.5	16	Right-sided headache	Complete palsy of the right cranial nerve III	Acute MRI: Thickening and enhancement of cranial nerve III on the right side	3 and 7 months later: reversal of abnormalities	OM
Mcmillan et al.	[20]	24	F	9	12	Right eye pain	Abrupt onset right pupil dilatation, diplopia, and ptosis	Acute MRI: an area of prominent thickening and contrast enhancement at the cisternal portion of the right oculomotor nerve	10 weeks later: resolution of abnormal enhancement of the right third cranial nerve, but persistent thickening of the previously enhancing segment	OM
Mcmillan et al.	[20]	25	M	1	1	Not described	Abrupt onset left ptosis and eye deviation	MRI: a small area of increased enhancement on the anterior surface of the left peduncle at the site of exit of the left oculomotor nerve	17 months later: enhancement and thickened of the cistern portion of left oculomotor nerve	OM
Mcmillan et al.	[20]	26	F	16	16	Throbbing, right-sided headache with photophobia	Right pupil dilatation, no change in visual acuity	MRI: normal	2 weeks later: definite enhancement of the cistern portion of the right oculomotor nerve	OM
Orssaud et al.	[28]	27	F	14	14	Supraorbital and left ocular pain in upward movements	Complete III nerve palsy	MRI: normal	N A	Tolosa–Hunt syndrome
Orssaud et al.	[28]	28	M	5	5	Migraine	Complete III nerve palsy	MRI: normal	Repeat MRI 2 years later (relapse): normal	OM
Vieria et al.	[37]	29	M	0.8	7	Frontotemporal and orbital pain always on the right side, photophobia, and phonophobia	Ptosis, external ophthalmoplegia and mydriasis	Infundibular dilatation of a perforating branch of the posterior cerebral artery adiacent to the III nerve	NA	OM
Arasho	[38]	30	M	5	15	Left hemicranial, more retro-orbital, throbbing/aching headache; photophobia; and phonophobia	Complete III nerve palsy	Acute MRI: no mass lesion	NA	OM
Borade et al.	[29]	31	F	5	6	Bilateral throbbing headache with photophobia and intollerance to loud sounds; vomiting	Ptosis on the right side and diplopia	Acute MRI: thickened and enhancing right oculomotor nerve in the suprasellar cistern region	NA	OM
Vecino-Lopez et al.	[30]	32	F	0.5	3	2 months later: headache, moderate intensity; episodes <24 hours	Right eye ptosis with mydriasis, complete III nerve palsy	MRI: enlargement and enhancement with contrast of the cisternal portion of the oculomotor nerve	Follow-up MRI: reduced enlargement and enhancement with contrast of the cisternal portion of the oculomotor nerve	OM
Miglio et al.	[39]	33	M	8	8	Headache on the right supraorbital side; photophobia; and vomiting	Ptosis, outward deviation of the right eye and diplopia	MRI: focal enlargement and marked enhancement in the cisternal portion of the third right cranial nerve at the root exit zone	3 months later: reduced thickening on the third right cranial nerve and resolution of the enhancement	OM
Lierly et al.	[40]	34	F	3	10	Ipsilateral, throbbing headache	Acute onset of right ptosis with lateral/inferior eye deviation	MRI: isolated enhancement of the cisternal segment of the right oculomotor nerve	NA	OM
Da Rocha et al.	[41]	35	M	—	5	Severe frontal headache	Right cranial nerve paresis	T1-weighted postcontrast MRI: typical focal thickening and enhancement of the proximal cisternal segment of the 3 cranial nerve	3 years later: persistent focal thickening without evident enhancement	OM
Gelfand et al.	[23]	36	M	5	19	Left periorbital, sharp, throbbing headache, photophobia, and nausea	Not reported	Acute MRI: enhancement of the cisternal portion of the third nerve	1 year later: normal	OM
Gelfand et al.	[23]	37	M	4	13	Right, diffuse, throbbing headache, photophobia, phonophobia, nausea, and vomiting	Not reported	Normal	Normal	OM
Gelfand et al.	[23]	38	F	9	16	Right frontal throbbing headache	Not reported	—	Normal	OM
Gelfand et al.	[23]	39	M	3	10	Left, periorbital, sharp headache, nausea, and vomiting	Not reported	Acute MRI thickening and enhancement of cisternal portion of third nerve	Nonacute MRI: persistent thickening but no enhancement	OM
Verma et al.	[24]	40	F	6	9	Right hemicranial, largely retro-orbital throbbing/pulsatile headache with photophobia, phonophobia, and vomiting	Drooping of right eyelid with difficulty in moving the eyeball and diplopia	Acute MRI: thickened, enhancing right oculomotor nerve in the cisternal segment	3 months later: resolution of oculomotor nerve enhancement	OM
Riahi et al.	[42]	41	F	3 episodes: 2008 2010 2011	12	Left side headache with vomiting	Left eye ptosis and diplopia	Two cerebral MRI (2008–2010) normal	3 years later (third episode): tissular mass in the cavernous sinus, suggesting a third nerve schawannoma	Schwannoma
Jibia et al.	[43]	42	F	7	13	Migraine with shimmering scotomas	Incomplete right ptosis with semi-midriasis, diplopia	MRI: a right nodular schwannoma located within the cisternal segment of the oculomotor nerve	2 months later: no changes (absence of neuroradiological variation)	Schwannoma
Hurd and Sabo	[27]	43	M	4	12	Severe, ipsilateral, pulsatile headache with photophobia, phonophobia, and nausea	Left eye ptosis, corneal injection, mydriasis, lacrimation, exotropia, and diplopia	Initial MRI without contrast: no abnormalities	MRI with contrast: enhancing lesion along the cisternal segment of the left oculomotor nerve	RPON

## Additional Points

*Clinical Implications*. (i) Put the focus on the still open debate on arguments pro and contra a migrainous background of RPON; (ii) underlying the important role of serial brain MRIs in the long-term follow-up of RPON; (iii) further longitudinal studies are needed to clarify pathophysiological relationship between RPON and schwannoma.
